# Ultra-rapid Idylla™ *EGFR* mutation screening followed by next-generation sequencing: An integrated solution to molecular diagnosis of non-small cell lung cancer

**DOI:** 10.3389/fonc.2023.1064487

**Published:** 2023-03-31

**Authors:** Tian Qiu, Fanshuang Zhang, Bo Zheng, Zitong Feng, Weihua Li, Hua Zeng, Lixia Chu, Jianming Ying

**Affiliations:** Department of Pathology, National Cancer Center/National Clinical Research Center for Cancer/Cancer Hospital, Chinese Academy of Medical Sciences and Peking Union Medical College, Beijing, China

**Keywords:** rapid detection, epidermal growth factor receptor, Idylla^TM^ *EGFR* assay, Chinese NSCLC patients, molecular diagnosis

## Abstract

**Background:**

Rapid profiling of the *EGFR* mutations is crucial to help clinicians choose the optimal treatment for patients with advanced/metastatic Non-Small Cell Lung Cancer (NSCLC). Unfortunately, current diagnostic techniques, including ARMS-PCR and NGS, generally require several days to deliver final results. This diagnostic delay may lead to treatment delays for patients who are worsening rapidly.

**Methods:**

This study introduced the ultra-rapid Idylla™ system for rapid, sensitive and specific identification of the *EGFR* mutations among Chinese NSCLC patients. Idylla™ *EGFR* Assay, an integrated cartridge running on the Idylla™ system, which can detect 51 *EGFR* mutations directly from Formalin-Fixed, Paraffin-Embedded (FFPE) samples within 2.5 hours, was used in this study. The sensitivity and specificity of the Idylla™ system were evaluated in comparison with ARMS-PCR or NGS using 95 clinical samples.

**Results:**

The Idylla™ system achieved a sensitivity of 97.6%, a specificity of 100%, and an overall concordance of 97.9% for 95 retrospective samples. When compared to ARMS-PCR, the Idylla™ system demonstrated high accuracy with an overall agreement of 97.1% (34/35), a sensitivity of 95.2% (20/21) (95% CI, 76.2% - 99.9%), and an estimated specificity of 100% (12/12) (95% CI, 76.8% - 100%) for 35 prospective samples.

**Conclusions:**

This Idylla system provides a rapid, accurate and simple approach for screening *EGFR* mutations, which can guide Tyrosine Kinase Inhibitors (TKI) treatment for NSCLC patients in a timely manner.

## Introduction

Over the past decade, the discovery of oncogenic driver mutations has greatly facilitated the development of targeted drugs. The Epidermal Growth Factor Receptor (*EGFR*) Tyrosine Kinase Inhibitors (TKIs) remain the mainstay of targeted therapy for Non-Small Cell Lung Cancer (NSCLC) because *EGFR* mutations occur in 50% of patients with lung adenocarcinomas in the Asian population (1–3). Exon 19 deletions and the L858R point mutation in exon 21 account for 85% of all *EGFR* mutations, and some less common alterations including L861Q, G719X, and S768I make up the remaining 10% (4–7). These mutations can affect patients’ response to TKIs such as erlotinib, gefitinib, afatinib, osimertinib, or dacomitinib (8–13). It should be noted that patients with exon 20 insertions are not sensitive to the first or second generation of *EGFR* TKIs (14, 15). Similarly, approximately 60% patients treated with erolotinib, gefitinib, or afatinib eventually develop resistance due to the appearance of the T790M point mutation (16, 17). Therefore, the NCCN guidelines recommended *EGFR* mutation status be determined in NSCLC patients prior to initiating TKI therapy (18). Immunotherapy has been incorporated into the first- and second-line treatment strategies for NSCLC. However, NSCLC patients with *EGFR* mutations show a poor response to anti-PD-1/PD-L1 treatment, which suggests that *EGFR* is involved in regulating the tumour microenvironment and inhibiting immunotherapy (19). Immunotherapy is not currently recommended by NCCN guidelines for patients with *EGFR*-mutant NSCLC.

The Amplification Refractory Mutation System-Polymerase Chain Reaction (ARMS-PCR) and Next-Generation Sequencing (NGS) are widely used in Chinese patients to determine *EGFR* mutations from Formalin-Fixed Paraffin-Embedded (FFPE) tissue samples (20, 21). Both of these approaches suffer from labor intensive procedures, including DNA isolation and library preparation. These processes require considerable staff training in laboratory skills, data interpretation and reporting. Moreover, NGS testing is often outsourced to independent clinical laboratories due to the highly complex bioinformatics analyses. Generally, the typical turnaround time in clinical practices is three to five days for ARMS-PCR and more than two weeks for NGS. This inevitably leads to the significant delays in the delivery of result. Therefore, these approaches are not suitable for acutely deteriorating patients who can barely afford any treatment delays (22). Identification of *EGFR* mutation status within 24 hours could reduce the time between diagnosis and optimal treatment. It is urgent need to develop an ultra-rapid automated platform to test for *EGFR* mutation in the field, which would allow for faster diagnosis and treatment for patients with *EGFR*-mutant NSCLC. The Idylla™ *EGFR* automated real-time PCR assay provides an integrated solution by combining DNA extraction, thermal cycling and fluorescence detection. This approach streamlines the process and reduces the overall turnaround time for *EGFR* mutation testing. According to protocol, 51 *EGFR* mutations could be detected simultaneously from FFPE samples in 2.5 hours with <10 minutes of hands-on time. The automated workflow and compact size make it easy to deploy in any situation, which is particularly important for lower tier hospitals, that lack of the platform for high complexity molecular testing. The Idylla™ *EGFR* system has been extensively validated in Caucasian patients with lung adenocarcinoma patients and received European Community (CE)-marked approval in 2017 (23–26). In this study, we focus on validating of the performance of the Idylla™ *EGFR* system in Chinese NSCLC patients. We also optimize and discuss the molecular diagnosis of advanced NSCLC by combining the rapid *EGFR* characterization by Idylla™ assay with genomic profiling by NGS.

## Methods

### Samples and study design

A total of 96 restrospective FFPE samples were collected and assessed using the Idylla™ *EGFR* Assay at the Department of Pathology, Cancer Hospital, Chinese Academy of Medical Sciences (CAMS). Samples with a histological diagnosis of NSCLC and a tumor cell content of ≥10% were deemed eligible for inclusion in the study. *EGFR* mutational status of these samples were assessed between April 2017 and August 2018 using either ARMS-PCR or NGS (Illumina platform). Mutations detected by NGS that were beyond the scope of the Idylla™ *EGFR* Assay were not included in the analysis. In case of discordance, samples were retested by the Idylla™ assay, and if the results remained inconsistent, ARMS-PCR and NGS (Ion Torrent platform) were repeated for confirmation. Idylla™ tests were also repeated for the discordant cases by increasing tissue input or manual enrichment of tumor cell content *via* macro-dissection. Another 35 prospective samples were collected and screened for *EGFR* mutations with the Idylla™ system afterwards. The results were then compared with those obtained using ARMS-PCR and NGS between June 2020 and September 2020. The study was approved by the Institute Review Board of the National Cancer Center/National Clinical Research Center for Cancer/Cancer Hospital, Chinese Academy of Medical Sciences and Peking Union Medical College. The methods were carried out in accordance with approved guidelines. The written informed consent was obtained from all patients. This study followed the ethical standards of the institutional and/or national research committee and the 1964 Helsinki declaration and its later amendments or comparable ethical guidelines.

### Idylla™ *EGFR* mutation assay

The Idylla™ *EGFR* Assay is an integrated cartridge with all sample processing buffers and PCR reagents pre-loaded. This assay is specifically designed to detect 51 mutations in exons 18–21 of the *EGFR* gene (**Supplementary Table 1**). For the 96 retrospective samples, a single 8 μm FFPE tissue section containing ≥10% neoplastic cells was added in the cartridge for each test, following the instruction for use of the Idylla™ *EGFR* Assay. For the 35 prospective samples, a single 8 μm FFPE tissue section was used for surgical samples, while three 8 μm FFPE sections were used separately for biopsy samples. In cases where the neoplastic cell content was lower than 10%, tissue sections were macro-dissected to enrich the sample. Tissue section was sandwiched between two layers of wetted filter paper and loaded directly into the cartridge. The cartridge was then inserted into the Idylla™ system. The system completes sample processing and real-time PCR automatically and reports result of mutations directly. In the Idylla™ *EGFR* Assay, the control is a wild-type *EGFR* sequence included in the assay cartridge, and the sample of interest is the DNA extracted from the patient’s tissue sample. The difference between the Cq (Cycle of Quantification) values of the control and the sample of interest (ΔCq) is used to determine the presence or absence of a mutation. If the ΔCq falls within the reference range, a mutant signal is considered valid and a mutation is identified, otherwise, the sample is considered *EGFR* mutation-negative.

### DNA preparation for ARMS-PCR/NGS confirmation

DNA was isolated using the QIAamp DNA FFPE Tissue Kit (Qiagen, CA, USA) and was quantified by the Qubit double-stranded DNA (dsDNA) HS assay kit on the Qubit 3.0 fluorometer (Thermo Fisher Scientific, NH, USA) following the manufacturer’s instructions.

### *EGFR* mutation test using ARMS-PCR

ARMS-PCR was carried out using the National Medical Product Administration (NMPA) approved Human *EGFR* Mutation Detection Kit (ACCB, Beijing, China). The kit is capable of detecting 44 mutations in *EGFR* exon 18-21, and some of the target mutations differ from those detected by Idylla™ *EGFR* Assay (**Supplementary Table 1**). In accordance with the Kit’s Instruction for Use, 15 ng of genomic DNA from each sample was used for each test. The PCR reaction was performed with the following parameters: initial denaturation at 95°C for 10 min, followed by 40 cycles of denaturation at 95°C for 15 s and annealing at 60°C for 1 min. Mutation subtypes were determined by analyzing the threshold count (Ct) values of the samples, where mutations were identified when the Ct value was ≤36, following the manufacturer’s instructions. If the Ct value was between 36 and 39, the test was repeated, and if the result remained within this range, the sample was considered a possible *EGFR* mutant.

### Targeted next-generation sequencing using Illumina NGS

DNA-based hybrid capture sequencing was carried out following the protocol as previously reported (27). Genomic DNA was first fragmented using Covaris M220, and then subjected to end repair and adaptor ligation. DNA fragments ranging from 200 and 400 bp were isolated using beads hybridized with a capture-probe panel targeting all exons in 56 cancer-related genes. Subsequently, sequencing libraries were generated after PCR amplification. Indexed libraries were pooled together and then sequenced on a NextSeq N550 platform (Illumina, San Diego, USA). Sequencing data were analyzed by GATK 3.2.

### Confirmation of *EGFR* mutational status using ion torrent NGS

Discrepancies in mutation status were resolved using the Ion Ampliseq Colon and Lung Cancer Panel on the Ion Torrent PGM platform (Thermo Fisher Scientific, NH, USA) following the protocol as previously described (28). Briefly, 10 ng of genomic DNA from each sample was PCR-amplified and then ligated to different barcodes to generate a library. The libraries were mixed and clonally amplified onto the IonSpheres (ISPs) for template preparation, and sequencing was carried out on a 318 chip using the Torrent Suite Software. Mutations were annotated through Torrent Variant Caller and viewed with Integrative Genomics Viewer. Mutations with a coverage depth of ≥1000 and a minor allele frequency (MAF) ≥5% were considered positive using the Torrent Variant Caller.

## Results

### Baseline characteristics of the patients

**Figure 1:**
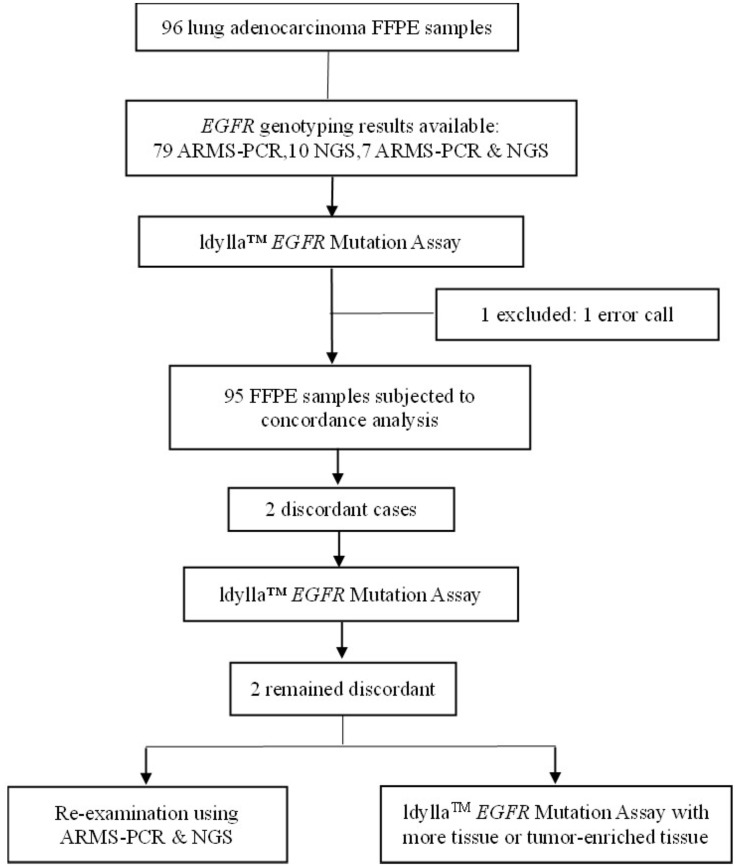
Flowchart of this testing strategy for Chinese NSCLC samples.

A total of 96 archival FFPE lung adenocarcinoma samples were included in this study for *EGFR* mutation analysis using the Idylla™ *EGFR* Assay as shown in **Figure 1**. Among the 96 samples, 79 were previously tested with AMRS-PCR, 11 with NGS, and 7 with both ARMS-PCR and NGS. Initially, 98.96% (95/96) of the sample were successfully tested by Idylla™ *EGFR*.(One result was invalid due to instrument error). Therefore, a total of 95 samples were included in the concordance analysis. Patients in the 95 samples had a median age of 61 years (interquartile range 37 to 81), with 62.1% (59/95) being female (**Supplementary Table 2**).

### Validation of the Idylla™ *EGFR* assay using retrospective samples

**Figure 2:**
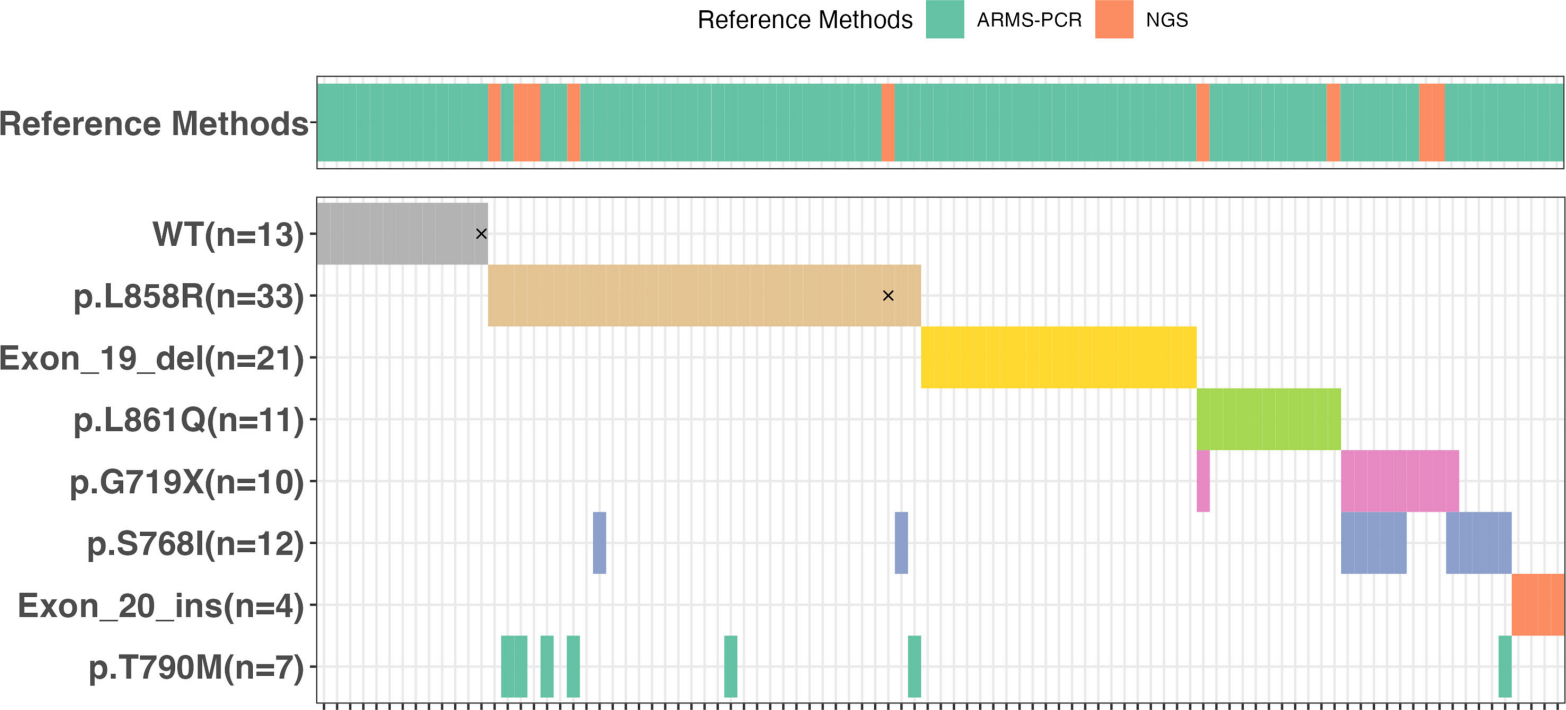
The oncoplot corresponding to *EGFR* mutations identified by Idylla™ assay in the 95 samples that were subjected to the reference methods ARMS-PCR or NGS. The discordances were marked by “×”.

Idylla™ detected mutations in 82 out of the 95 samples as presented in **Supplementary Table 3** and **Figure 2**. Of these mutated samples, 66 had a single mutation while 16 had two mutations, resulting in a total of 98 mutations being discovered by Idylla™. Among the 66 samples with a single mutation, there were 21 with Exon 19 deletion, 25 with L858R, and 4 with Exon 20 insertion mutations, accounting for 75.8% (50/66) of all mutations. For the remaining 16 samples, 10 had L861Q, 3 had G719X, and 3 had S768I mutations. The 16 samples with two mutations comprised 6 with G719X and S768I, 6 with L858R and T790M, 2 with L858R and S768I, 1 with G719X and L861Q, and 1 with T790M and S768I mutations. The frequency of different types of mutations, from high to low, is as follows: L858R at 33.7% (33/98), Exon 19 del at 21.4% (21/97), L861Q at 11.2% (11/98), G719X and S768I both at 10.2% (10/98), T790M at 6.1% (6/98), and Exon 20 ins at 4.1% (4/98). Among these 95 samples, only two exhibited inconsistent results between the Idylla™ *EGFR* and the reference method. In one sample (Sample No. 91#), Idylla failed to detect any mutation, whereas the reference method identified G719X + S768I mutations (NGS also detected E709K in this sample, which falls outside the detection range of the Idylla™). In the other sample (Sample No. 88#), Idylla detected only the L858R mutation, while the reference method revealed the presence of both L858R and L861Q mutations. Among all 101 mutations detected by the reference method, Idylla™ missed a total of 3 mutations in 2 samples. The Idylla™ system achieved a sensitivity of 97.6%, a specificity of 100%, and an overall concordance of 97.9%.

### Evaluation of the discordant cases

**Figure 3:**
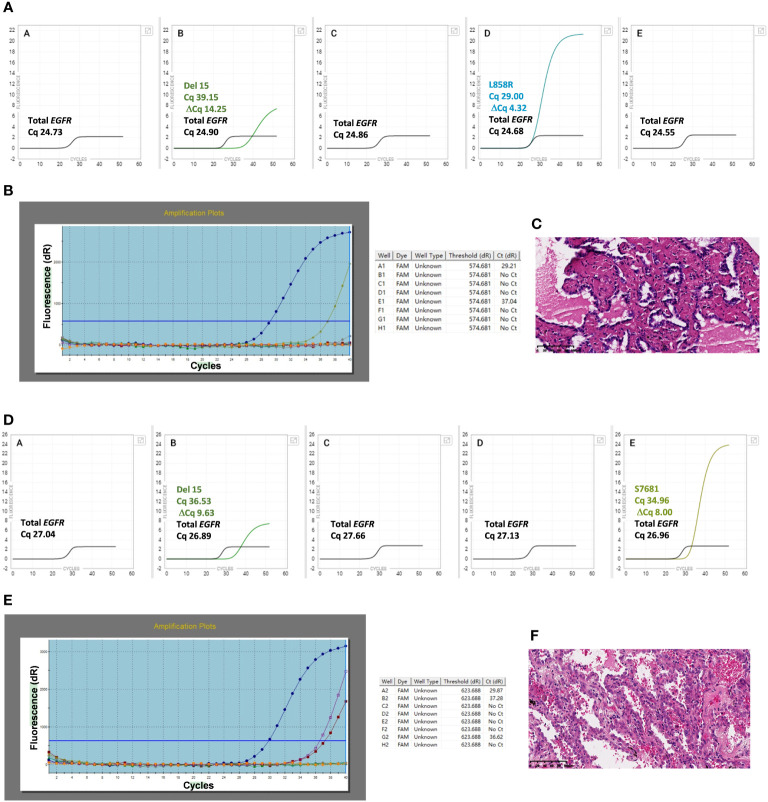
Idylla™ Explore version 2.5.1294.1 (Biocartis, Mechelen, Belgium) default display showing the detail of PCR curves and cycle of quantification (Cq) values of sample 88# **(3A)** and samples 91# **(3D)**. Cq values are label for the sample processing controls, *EGFR* Total, and the target for which a signal has been detected (default view). Panels A to E in the image represent the five PCR chambers in the Idylla™ cartridge. Details of PCR curves and Cycle threshold (Ct) values by ARMS-PCR for samples 88# **(3B)** and 91# **(3E)**. Ct value of well A is available for the quality control of DNA input, and Ct values of the other wells are for the target sequences detected. The H&E photos of samples 88 and 91 are displayed in **(3C, 3F)**.

The two discordant cases (91# and 88#) were re-examined using the Idylla™ assay, followed by Ion Torrent NGS and ARMS-PCR (**Table 1**) The H&E photos of samples 88 and 91 were displayed in **Figure 3C, F**. Sample 91# was wild-type when re-tested with Idylla™ using only one FFPE section. However, when the number of FFPE sections was increased to two, S768I mutation was detected. Further increasing the number of sections to three or four, both G719X and S768I mutations were identified as shown in **Table 2**. In the second Idylla™ test of Sample 88#, the same result was obtained as in the first test, with only L858R mutation detected. Subsequently, the neoplastic cell content of the FFPE sections was enriched through macro-dissection, and 2-4 sections were re-tested with Idylla™. However, the result remained the same for 88#, with only the L858R mutation being detected. As illustrated in **Figure 3D**, the median Cq value for sample 91# was 27.04, indicating that the amount of amplifiable DNA in the cartridge was less than 1.584 ng according to the manufacturer’s instructions. For sample 88#, the median Cq value for the *EGFR* control was 24.73 (**Figure 3A**), which suggested that the amount of amplifiable DNA in the cartridge was between 7.92 ng and 15.84 ng. However, most of the other samples had a median Cq value of less than 20.00 for the *EGFR* control, which corresponded to more than 396 ng of amplifiable DNA in the cartridge. The Ct values for samples 88# and 91# by ARMS-PCR were 29.21 and 29.87, respectively, which were close to the upper limit of detection of the assay. Moreover, the Ct values of samples 88# and 91# by ARMS-PCR were 37.04 for L858R (**Figure 3B**), 37.28 for G719X, and 36.62 for S768I (**Figure 3E**), suggesting that the discordance was caused by low DNA input or low mutational allele frequency.

**Table 1:** Discordant cases between the Idylla™ *EGFR* assay and reference methods in the 95 retrospective samples.

Sample	Sample type	Tumor content	Surface area	Reference methods	Results by reference methods	Idylla™ initial result	Idylla™ retest result	ARMS-PCR confirmation	Ion Torrent NGS confirmation
88#	Surgical	10%	1.5cm^2^	Illumina NGS	E709K/L858R/L861Q (4.8%/3.3%/3.4%)	L858R	L858R	L858R	L861Q (7.2%)
91#	Surgical	40%	0.25cm^2^	ARMS-PCR	G719X/S768I	Wild-type	Wild-type	G719X/S768I	G719C/S768I (14.2%/13.1%)

**Table 2:** The impact of tumor cell enrichment and increasing sample input on the performance of Idylla™ *EGFR* assay.

Sample	Tumor content	Surface area	Tumor cell enrichment	Results by reference methods	Initial Idylla™ result	Number of tissue sections and results of Idylla™ *EGFR* retesting			
						1	2	3	4
88#	10%	1.5cm^2^	Yes	E709K/L858R/L861Q (4.8%/3.3%/3.4%)	L858R	L858R	L858R	L858R	L858R
91#	40%	0.25cm^2^	No	G719X/S768I	Wild-type	\	S768I	G719X/S768I	G719X/S768I

### Combination of Idylla test with NGS to optimizing molecular diagnosis of NSCLC

Thirty-five prospective samples were tested using the Idylla™ *EGFR* Assay in parallel with ARMS-PCR and NGS. Of the 35 samples, 23 were biopsy tissue samples and 12 were surgical tissue samples. Out of the 23 biopsy tissue samples, 2 had a neoplastic cell content of 10%, which is at the minimum threshold required for the Idylla sample input. Patients in the 35 samples had a median age of 58 years (interquartile range 42 to 84), with 54.3% (19/35) being female (**Supplementary Table 2**). Eleven patients were untreated, four had undergone chemotherapy, and three had received or were currently undergoing *EGFR*-TKIs. The treatment status of the remaining seventeen patients was unknown. Idylla™ detected mutations in 21 out of the 35 samples, resulting in positive rate of 60% (21/35) (**Supplementary Table 4** **)**. Of these mutated samples, 15 had a single mutation while 5 had two mutations, resulting in a total of 25 mutations being discovered by Idylla™. Among the 15 samples with a single mutation, there were 6 with Exon 19 deletion, 8 with L858R, and 1 with G719X mutations. Each of the remaining 6 samples had a distinct combination of two mutations. The frequency of different types of mutations, from high to low, is as follows: L858R at 44.0% (11/25), Exon 19 del at 32.0% (8/25), G719X, S768I and T790M each at 8.0% (2/27). Among these 35 samples, only 1(Sample No. 2#) exhibited inconsistent results between the Idylla™ *EGFR* and ARMS-PCR. Idylla detected only the L858R mutation in 2#, while ARMS-PCR revealed the presence of both L858R and T790M mutations. The Cq value of 2# in Idylla was above 26(data not shown). Two samples were found to have the 19Del variant according to NGS, but they were reported as wild-type by both Idylla™ and ARMS-PCR (**Table 3**), as both variant types fell outside the detection range of the two methods. Additionally, the presence of C797S in cis with T790M mutation was identified by NGS in one sample. Compared to ARMS-PCR, the Idylla™ system demonstrated high accuracy with an overall agreement of 97.1% (34/35), a sensitivity of 95.2% (20/21) (95% CI, 76.2%-99.9%), and an estimated specificity of 100% (12/12) (95% CI, 76.8%-100%). When compared to NGS, including the two rare 19Del variations, the overall accuracy was 91.4% (32/35), with a sensitivity of 87% (20/23) (95% CI, 66.4%-97.2%), and a specificity of 100% (12/12) (95% CI, 73.5%-100%).

**Table 3:** Discordant cases between the Idylla™ *EGFR* assay and reference methods in the 35 routine clinical samples.

Sample	Sample type	Tumor content	Idylla™ *EGFR*	ARMS-PCR	Illumina NGS *EGFR*	Illumina NGS other genes
21#	biopsy	20%	L858R	L858R/T790M	L858R/T790M/C797S	\
24#	biopsy	40%	Wild-type	Wild-type	19Del (c.2240_2259>CT,PL747_PL753>S)(20.5%)	\
25#	biopsy	50%	Wild-type	Wild-type	19Del (c.2251_2276>TC, p.T751_I759>S)(8.1%)	TP53

## Discussion

In this study, we evaluated the performance of the ultra-rapid Idylla™ system for the rapid, sensitive, and specific identification of *EGFR* mutations in Chinese NSCLC patients. The Idylla™ system exhibited a sensitivity of 97.6%, a specificity of 100%, and an overall concordance of 97.9% for 95 retrospective samples. Additionally, when compared to ARMS-PCR, the Idylla™ system demonstrated high accuracy with an overall agreement of 97.1% (34/35), a sensitivity of 95.2% (20/21) (95% CI, 76.2% - 99.9%), and an estimated specificity of 100% (12/12) (95% CI, 76.8% - 100%) for 35 prospective samples.

Out of the 95 retrospective samples, only two samples showed discordant results between the Idylla™ *EGFR* and the reference method. One of the discrepancies (91#) was resolved by increasing the sample input with additional tissue sections. Further analysis revealed that the tissue area of 91# was only 0.25 cm^2^, and the Cq value analysis showed that the amount of amplifiable DNA in the sample after extraction was only 1.584 ng, indicating that insufficient sample volume was the main reason for the inconsistent result. The Cq value of the only discrepant result among the 35 prospective samples also indicated the same. The Idylla *EGFR* Assay does not specify the minimum tissue area for loading, but only requires the tumor cell proportion and the maximum tissue area for loading. However, the lack of a minimum tissue area requirement may lead to missed or erroneous results. Despite enriching the neoplastic cell content and increasing the tissue sections, sample 88# still exhibited discordant results. Further analysis revealed that allele frequency of the L861Q mutation, missed in the Idylla assay, was 3.4% according to NGS result. This indicates that Idylla has lower sensitivity than NGS for detecting L861Q mutations with low allele frequency.

**Figure 4:**
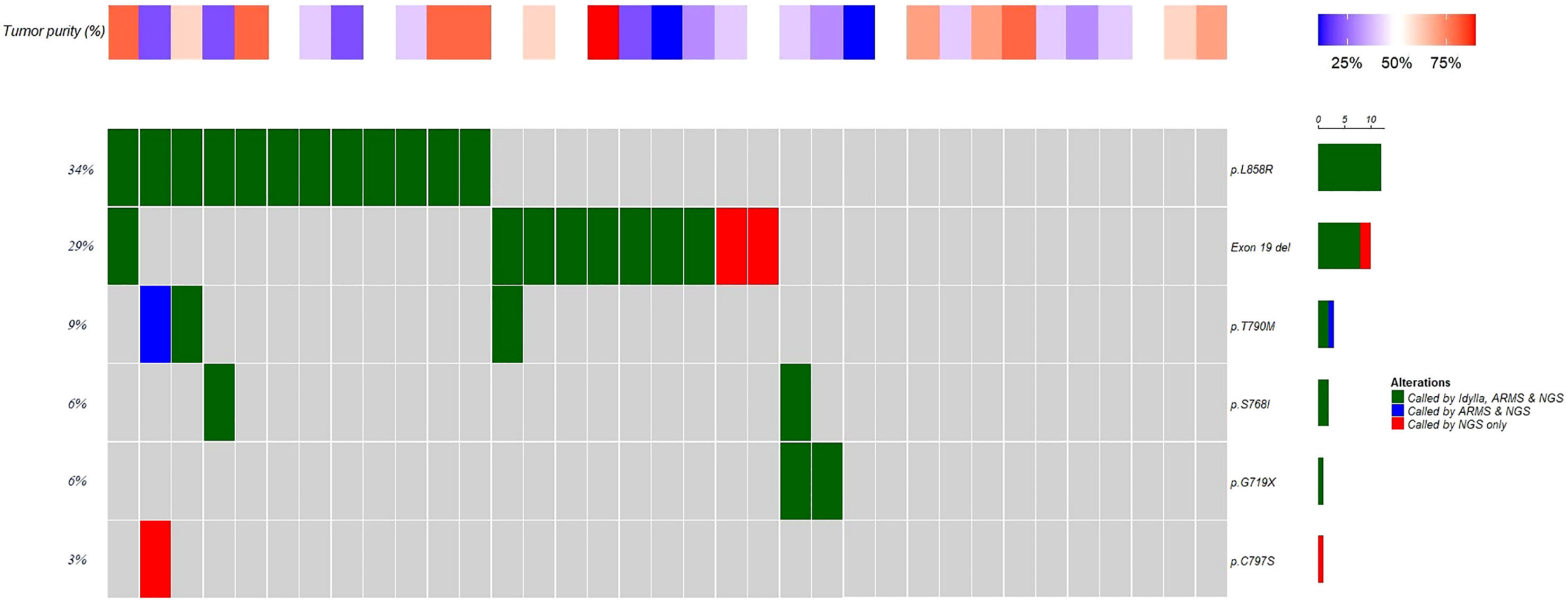
Analysis results of 35 clinical samples with Idylla, ARMS-PCR and NGS.

Among the 95 retrospective samples, NGS detected an additional E709K mutation in one sample. In the 35 prospective samples, NGS detected two rare 19 deletion mutations in two samples and an additional C797S cis mutation in one sample. In addition, in five samples with *EGFR* mutations, NGS detected PIK3CA mutations in two samples and TP53 mutations in three samples (**Figure 4**). In 12 samples with wild-type *EGFR* analyzed in this study, NGS detected nine samples with mutations in other genes related to tumorigenesis, including four with KRAS mutations, two with HER2 mutations, one with an EML4-ALK fusion mutation, and three with TP53 mutations (data not shown). This indicates that NGS has a significant advantage over traditional fluorescence-based quantitative PCR methods in terms of panel size. This may provide additional benefits to patients, such as those with HER2 exon 20 mutations and KRAS G12C mutations. Despite this, AMRS-PCR and Idylla *EGFR* still detect the majority of clinically validated *EGFR* mutations that can provide clinical benefits to patients. Compared to AMRS-PCR, Idylla *EGFR* can detect more types of *EGFR* mutations (51 versus 44).

**Figure 5:**
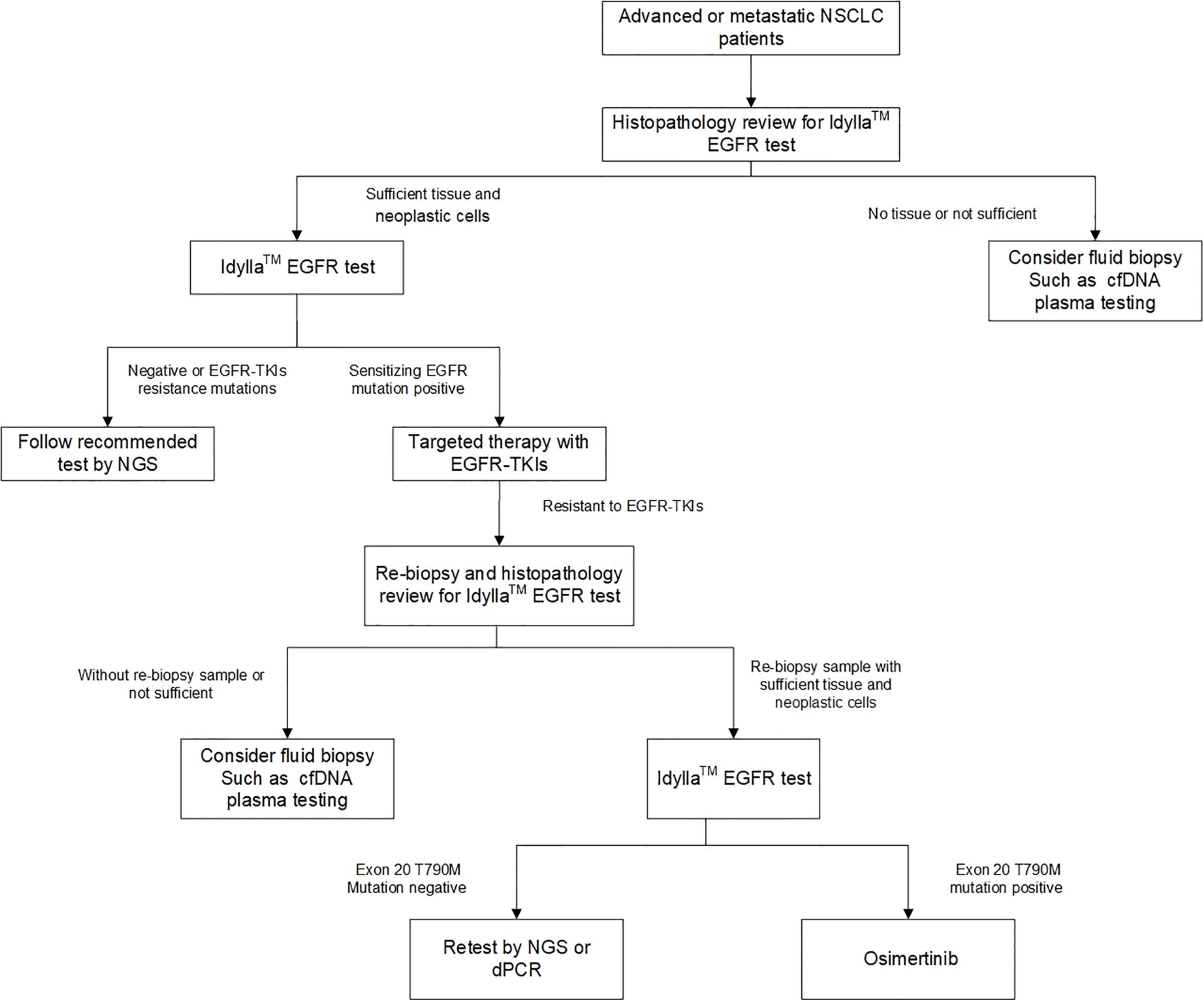
An optimized workflow for molecular diagnosis of NSCLC by combining rapid *EGFR* detection with genomic profiling by NGS.

The Idylla system is a fully automated PCR testing system that follows a “sample in, result out” approach, offering advantages such as speed, low sample volume requirement, and standardized testing process. The use of this system eliminates the need for sample pooling and enables on-demand testing, leading to improved efficiency of testing equipment utilization. In this study, 93 out of 95 retrospective samples yielded consistent results in the first test using a single FFPE slice. Among the 35 prospective samples, 12 surgical samples yielded consistent results in the first test using a single FFPE slice. Only one out of 21 biopsy samples using three FFPE slices showed inconsistent results in the first test. The low sample volume requirement expands the accessibility of the Idylla *EGFR* assay and benefits more patients. The hands-on time of the Idylla *EGFR* assay is less than 2 minutes, and the turnaround time from sample input to result output is less than 2.5 hours, with automatic report sending. In the prospective study, the turnaround time for different testing methods was compared. The average time from detection to report sending was 3-5 working days for ARMS-PCR, 10-15 working days for NGS, and 2 working days for Idylla *EGFR*. Based on this, we proposed an optimized flow for non-small cell lung cancer molecular diagnosis (**Figure 5**) as a supplement to routine molecular diagnosis. In this flow, Idylla *EGFR* is first deployed to test emergency patients first. If the result is negative, NGS is used to detect other potential gene mutations that may benefit the patient. If the result is positive, based on the patient’s pathology and staging diagnosis, first- or second-generation *EGFR* TKIs such as gefitinib, erlotinib, dacomitinib, and osimertinib can be used. If the tissue or biopsy sample is insufficient, liquid biopsy can be used for testing. Based on the mutation detection results of the 130 cases (95 retrospective and 35 prospective) in this study, this flow enabled 97.7% (127/130) of patients to receive timely treatment after the first use of Idylla *EGFR*.

## Conclusion

In conclusion, the Idylla™ *EGFR* mutation system provides an ultra-rapid, accurate, and easy to-use automated solution for molecular genotyping. Integrating this ultra-rapid detection system as a critical screening step with NGS could provide timely and comprehensive benefits to patients, ultimately leading to better treatment outcomes.

## Data availability statement

The datasets presented in this study can be found in online repositories. The names of the repository/repositories and accession number(s) can be found below: BioProject, accession number PRJNA923137.

## Ethics statement

The studies involving human participants were reviewed and approved by the Institute Review Board of the National Cancer Center/National Clinical Research Center for Cancer/Cancer Hospital, Chinese Academy of Medical Sciences and Peking Union Medical College. The patients/participants provided their written informed consent to participate in this study.

## Author contributions

(I) Conception and design: TQ, JY. (II) Administrative support: JY. (III) Provision of study materials or patients: TQ, FZ. (IV) Collection and assembly of data: TQ, FZ. (V) Data analysis and interpretation: TQ, FZ, BZ, ZF, WL, HZ, LC. (VI) Manuscript writing: All authors. All authors contributed to the article and approved the submitted version.
