# Supplementary material for: Ultra-rapid Idylla™ EGFR mutation screening followed by next-generation sequencing: An integrated solution to molecular diagnosis of non-small cell lung cancer
Source: Front Oncol. 2023 Mar 31;13:1064487. doi: 10.3389/fonc.2023.1064487 (PMC10102514; doi:10.3389/fonc.2023.1064487)
Supplement: Supplementary file 1 [file DataSheet_1.docx]

**Supplementary Table 1. Mutations detected by the Idylla^TM^ *EGFR* Mutation Assay and the ARMS-PCR *EGFR* Mutation Assay**

|  | **Idylla^TM^ *EGFR* Mutation Assay** | | | **ARMS-PCR *EGFR* Mutation Assay** | | | |
| --- | --- | --- | --- | --- | --- | --- | --- |
| **Exon** | **Nucleotide change** | **Protein change** | **Genotype call** | **Exon** | **Nucleotide change** | **Protein change** | **Cosmic ID** |
| 18(4) | c.2156G>C | p.Gly719Ala | G719A/C/S | 18(3) | 2156G>C | G719A | 6239 |
|  | c.2155G>A | p.Gly719Ser |  |  | 2155G>A | G719S | 6252 |
|  | c.2155G>T | p.Gly719Cys |  |  | 2155G>T | G719C | 6253 |
|  | c.2154_2155delinsTT | p.Gly719Cys(2) |  |  | / | / | / |
| 19(36) | c.2238_2248delinsGC | p.Leu747_Ala750delinsPro | Exon 19 deletion | 19(31) | 2238_2248>GC | L747_A750>P | 12422 |
|  | c.2239_2248delinsC |  |  |  | 2239_2248>C | L747_A750>P | 12382 |
|  | c.2240_2248del | p.Leu747_Ala750delinsSer |  |  | / | / | / |
|  | c.2239_2247del | p.Leu747_Glu749del |  |  | 2239_2247del9 | L747_E749del | 6218 |
|  | c.2239_2251delinsC | p.Leu747_Thr751delinsPro |  |  | 2239_2251>C | L747_T751>P | 12383 |
|  | c.2240_2251del | p.Leu747_Thr751delinsSer |  |  | 2240_2251del12 | L747_T751>S | 6210 |
|  | c.2235_2249del | p.Glu746_Ala750del |  |  | 2235_2248>AATTC | E746_A750>IP | 13550 |
|  | c.2236_2250del |  |  |  | 2236_2248>CAAC | E746_A750>QP | 13557 |
|  | c.2239_2253del | p.Leu747-Thr751del |  |  | 2239_2253del15 | L747_T751del | 6254 |
|  | c.2240_2254del |  |  |  | 2240_2254del15 | L747_T751del | 12369 |
|  | c.2238_2252del |  |  |  | 2238_2252>GCA | L747_T751>Q | 12419 |
|  | c.2237_2251del | p.Glu746_Thr751delinsAla |  |  | 2237_2251del15 | E746_T751>A | 12678 |
|  | c.2235_2252delinsAAT | p.Glu746_Thr751delinsIle |  |  | 2235_2252del18 | E746_T751del | 24869 |
|  | c.2237_2252delinsT | p.Glu746_Thr751delinsVal |  |  | / | / | / |
|  | c.2234_2248del | p.Lys745_Ala750delinsThr |  |  | 2233_2247del15 | K745_E749del | 26038 |
|  | c.2236_2253delinsCTA | p.Glu746_Thr751delinsLeu |  |  | 2236_2253del18 | E746_T751del | 12728 |
|  | c.2237_2253delinsTA | p.Glu746_Thr751delinsVal |  |  | 2235_2251>AATTC | E746_T751>IP | 13552 |
|  |  |  |  |  | 2235_2252>AAT | E746_T751>I | 13551 |
|  | c.2235_2251delinsAG | p.Glu746_Thr751delinsAla |  |  | 2235_2251>AG | E746_T751>A | 13549 |
|  | c.2236_2253delinsCAA | p.Glu746_Thr751delinsGln |  |  | / | / | / |
|  | c.2230_2249delinsGTCAA | p.Ile744_Ala750delinsValLys |  |  | / | / | / |
|  | c.2240_2257del | p.Leu747_Pro753delinsSer |  |  | 2240_2257del18 | L747_P753>S | 12370 |
|  | c.2237_2255delinsT | p.Glu746_Ser752delinsVal |  |  | 2237_2255>T | E746_S752>V | 12384 |
|  | c.2239_2256del | p.Leu747_Ser752del |  |  | 2239_2256del18 | L747_S752del | 6255 |
|  | c.2236_2253del | p.Glu746_Thr751del |  |  | 2239_2252>CA | L747_T751>Q | 12420 |
|  | c.2239_2258delinsCA | p.Leu747_Pro753delinsGln |  |  | 2239_2258>CA | L747_P753>Q | 12387 |
|  | c.2237_2254del | p.Glu746_Ser752delinsAla |  |  | 2237_2254del18 | E746_S752>A | 12367 |
|  | c.2238_2255del | p.Glu746_Ser752delinsAsp |  |  | 2238_2255del18 | E746_S752>D | 6220 |
|  | c.2237_2257delinsTCT | p.Glu746_Pro753delinsValSer |  |  | / | / | / |
|  | c.2236_2255delinsAT | p.Glu746_Ser752delinsIle |  |  | 2235_2246del12 | E746_E749del | 28517 |
|  | c.2236_2256delinsATC |  |  |  | 2236_2248>AGAC | E746_A750>RP | 12413 |
|  | c.2237_2256delinsTC | p.Glu746_Ser752delinsVal |  |  | 2237_2256>TC | E746_S752>V | 18426 |
|  | c.2237_2256delinsTT |  |  |  | / | / | / |
|  | c.2235_2255delinsGGT |  |  |  | 2235_2255>AAT | E746_S752>I | 12385 |
|  | c.2238_2258del | p.Leu747_Pro753del |  |  | 2238_2252del15 | L747_T751del | 23571 |
|  | c.2236_2256del | p.Glu746_Ser752del |  |  | 2236_2250del15 | E746_A750del | 6225 |
|  | c.2253_2276del | p.Ser752_Ile759del |  |  | 2235_2249del15 | E746_A750del | 6223 |
| 20(7) | c.2369C>T | p.Thr790Met | T790M | 20(8) | 2369C>T | T790M | 6240 |
|  | c.2303G>T | p.Ser768Ile | S768I |  | 2303G>T | S768I | 6241 |
|  | c.2310_2311insGGT | p.Asp770_Asn771insGly | Exon 20 insertion |  | 2307_2308insGCCAGCGTG | V769_D770insASV | 12376 |
|  | c.2308_2309insGCCAGCGTG | p.Val769_Asp770insAlaSerVal |  |  | 2309_2310AC>CCAGCGTGGAT | V769_D770insASV | 13558 |
|  | c.2308_2311delinsCCAGCGTGGAT | p.Val769_Asp770insAlaSerVal |  |  | 2311_2312insGCGTGGACA | D770_N771insSVD | 13428 |
|  | c.2311_2312insGCGTGGACA | p.Asp770_Asn771insSerValAsp |  |  | 2310_2311insGGT | D770_N771insG | 12378 |
|  | c.2319_2320insCAC | p.His773_Val774insHis |  |  | 2319_2320insCAC | H773_V774insH | 12377 |
|  | / | / |  |  | 2319_2320insAACCCCCAC | H773_V774insNPH | 12381 |
| 21(4) | c.2573T>G | p.Leu858Arg | L858R | 21(3) | 2573T>G | L858R | 6224 |
|  | c.2573_2574delinsGT |  |  |  | 2573_2574TG>GT | L858R | 12429 |
|  | c.2573_2574delinsGA |  |  |  | / | / | / |
|  | c.2582T>A | p.Leu861Gln | L861Q |  | 2582T>A | L861Q | 6213 |

Note: The different mutation that tested by Idylla and ARMS-PCR are highlighted with red font.

**Supplementary Table 2**. **Baseline characteristics of the retrospective cohort (n=95) and prospective cohort (n=35)**

| **retrospective cohort (n=95)** | | |
| --- | --- | --- |
| **Median age, y (Min, Max)** | | 61（37-81） |
| **Sex, n** | **Male** | 36 |
|  | **Female** | 59 |
| **Tumor stage, n** | **I** | 65 |
|  | **II** | 13 |
|  | **III** | 13 |
|  | **IV** | 3 |
|  | **Unknown** | 1 |
| **Smoking history, n** | **Smoker** | 23 |
|  | **Non-smoker** | 72 |
| **Tumor site, n** | **Primary** | 88 |
|  | **Metastatic** | 3 |
|  | **Unknown** | 4 |
| **Sample type, n** | **Surgical** | 93 |
|  | **Biopsy** | 2 |
| **Treatment, n** | **Treatment naive** | 91 |
|  | **TKIs/chemotherapy** | 4 |
| **prospective cohort (n=35)** | | |
| **Median age, y (Min, Max)** | | 58（42-84） |
| **Sex, n** | **Male** | 16 |
|  | **Female** | 19 |
| **Tumor stage, n** | **I** | 8 |
|  | **II** | 2 |
|  | **III** | 3 |
|  | **IV** | 4 |
|  | **Unknown** | 18 |
| **Smoking history, n** | **Smoker** | 6 |
|  | **Non-smoker** | 15 |
|  | **Unknown** | 14 |
| **Sample type, n** | **Surgical** | 12 |
|  | **Biopsy** | 23 |
| **Treatment, n** | **Treatment naive** | 11 |
|  | **Chemotherapy** | 4 |
|  | **EGFR-TKIs** | 3 |
|  | **Unknown** | 17 |

**Supplementary Table 3**. **Idylla^TM^ *EGFR* genotyping results in comparison with the results of reference methods in the retrospective samples (n=95)**

|  |  | **Reference methods (ARMS-PCR or NGS** | | | | | | | | | | | | | |
| --- | --- | --- | --- | --- | --- | --- | --- | --- | --- | --- | --- | --- | --- | --- | --- |
|  |  | **WT** | **Exon19 del** | **p.L858R** | **p.L861Q** | **p.G719X** | **p.768I** | **Exon20 ins** | **p.G719X;p.L861Q** | **p.719X; p.768I** | **p.L858R; p.768I** | **p.L858R;p.L861Q;p.E709K** | **p.L858R;p.T790M** | **p.T790M;p.768I** | **Total** |
| **Idylla** | **WT** | 12 |  |  |  |  |  |  |  |  |  |  |  |  | 13 |
|  | **Exon19 del** |  | 21 |  |  |  |  |  |  |  |  |  |  |  | 21 |
|  | **p.L858R** |  |  | 24 |  |  |  |  |  |  |  | 1 |  |  | 25 |
|  | **p.L861Q** |  |  |  | 10 |  |  |  |  |  |  |  |  |  | 10 |
|  | **p.G719X** |  |  |  |  | 3 |  |  |  |  |  |  |  |  | 3 |
|  | **p.768I** |  |  |  |  |  | 3 |  |  |  |  |  |  |  | 3 |
|  | **Exon20 ins** |  |  |  |  |  |  | 4 |  |  |  |  |  |  | 4 |
|  | **p.G719X;p.L861Q** |  |  |  |  |  |  |  | 1 |  |  |  |  |  | 1 |
|  | **p.719X;p.768I** |  |  |  |  |  |  |  |  | 6 |  |  |  |  | 6 |
|  | **p.L858R;p.L861Q;p.E709K** |  |  |  |  |  |  |  |  |  | 2 |  |  |  | 2 |
|  | **p.L858R;p.T790M** |  |  |  |  |  |  |  |  |  |  |  | 6 |  | 6 |
|  | **p.T790M;p.768I** |  |  |  |  |  |  |  |  |  |  |  |  | 1 | 1 |

**Supplementary Table 4**. **Idylla^TM^ *EGFR* genotyping results in comparison with the results of reference methods in the prospective samples (n=35)**

|  | | **Reference methods (ARMS-PCR and NGS)** | | | | | | | | | |
| --- | --- | --- | --- | --- | --- | --- | --- | --- | --- | --- | --- |
|  |  | **WT** | **Exon19 del** | **p.L858R** | **p.G719X** | **Exon19 del;p.T790M** | **Exon19 del;p.L858R** | **p.L858R; p.768I** | **p.L858R;p.T790M** | **p.G719X;p.768I** | **Total** |
| **Idylla** | **WT** | 12 | 2 |  |  |  |  |  |  |  | 14 |
|  | **Exon19 del** |  | 6 |  |  |  |  |  |  |  | 6 |
|  | **p.L858R** |  |  | 8 |  |  |  |  |  |  | 9 |
|  | **p.G719X** |  |  |  | 1 |  |  |  |  |  | 1 |
|  | **Exon19 del;p.T790M** |  |  |  |  | 1 |  |  |  |  | 1 |
|  | **Exon19 del;p.L858R** |  |  |  |  |  | 1 |  |  |  | 1 |
|  | **p.L858R; p.768I** |  |  |  |  |  |  | 1 |  |  | 1 |
|  | **p.L858R;p.T790M** |  |  |  |  |  |  |  | 1 |  | 1 |
|  | **p.G719X;p.768I** |  |  |  |  |  |  |  |  | 1 | 1 |
